# Biochemical and agro-morphological traits-based mining for Malt Barley Germplasm

**DOI:** 10.3389/fnut.2025.1480708

**Published:** 2025-02-18

**Authors:** Shakti Khera, Prakash Kumar, Shashi Bhushan Tripathi, Sherry Racheal Jacob, Dinesh Kumar, Charan Singh, Rakesh Bhardwaj, Sudhir Pal Ahlawat, Jai Chand Rana, Amritbir Riar

**Affiliations:** ^1^ICAR-National Bureau of Plant Genetic Resource, Pusa, New Delhi, India; ^2^TERI School of Advanced Studies, Vasant Kunj, New Delhi, India; ^3^ICAR-Indian Agricultural Statistics Research Institute, Pusa, New Delhi, India; ^4^ICAR-Indian Institute of Wheat and Barley Research, Karnal, Haryana, India; ^5^The Alliance of Bioversity International & CIAT-India Office, NASC Complex, New Delhi, India; ^6^Research Institute of Organic Agriculture (FiBL), Frick, Switzerland

**Keywords:** protein, starch, amylose, beta glucan, phenols, yield contributing traits

## Abstract

Barley serves as a crucial feed crop and is also utilized for baking, malting, and brewing purposes. In India, the consistent demand for malting-type barley has not been met due to the lack of suitable varieties. This study evaluated 136 barley germplasm accessions for diversity in biochemical and agro-morphological traits. The accessions were grown in Augmented Block Design and biochemical estimations were carried out using official and standard methods. The accessions exhibited substantial biochemical diversity with protein content (PC) ranging from 8.6 to 17.4%, starch content (SC) from 32.4 to 60.3%, amylose content (AC) from 13.3 to 19.3%, *β*-glucan content (βgC) from 1.31 to 6.06%, and total phenol content (TPC) from 8.6 to 17.4%. The agro-morphological traits also displayed considerable variation, with days to spike emergence (DSE) ranging from 74 to 124 days, days to physiological maturity (DPM) from 119 to 147 days, plant height (PH) from 82 to 165 cm, spike length (SL) from 5 to 11 cm, spikelet triplet groups (STG) per spike from 13 to 35, grain number per spike (GNS) from 15 to 71, hundred-grain weight (HGW) from 2 to 6.7 grams, and grain yield per meter row (GY) from 13.7 to 236.3 grams. Multivariate analyses, including the Mantel test, Pearson’s correlation, principal component analysis (PCA), and hierarchical cluster analysis (HCA), were conducted. No significant correlation was observed between biochemical and agro-morphological traits. However, significant positive correlations were found between SC and AC (*r* = 0.48) and βgC with PC (*r* = 0.2). Significant negative correlations were observed between SC and PC (*r* = −0.41) and AC with PC (*r* = −0.4). Highly significant positive correlations were observed between DSE and DPM (*r* = 0.55), GY and PH (*r* = 0.29), PH and DPM (*r* = 0.2), and HGW and SL (*r* = 0.25). The GWL was significantly positively correlated with βgC (*r* = 0.257) and significantly negatively correlated with TPC (*r* = −0.235). Apart from Grain Width to Length Ratio (GWL), no other agro-morphological trait was significantly correlated with any biochemical trait. Various accessions suitable for malting, human food, and cattle feed applications were identified.

## 1 Introduction

Barley (*Hordeum vulgare* L.), originating from the Abyssinian center of diversity, is one of the earliest domesticated crops in the world. Revered for its adaptability and versatility, barley has been used historically as livestock and poultry feed, as a dietary staple, and as a key ingredient in brewing alcoholic beverages (1). The crop’s dual utility for food and industrial purposes emphasizes its economic importance globally and in regions like India, where distinct types of barley are cultivated. Barley exists in two primary forms based on spike morphology: six-rowed and two-rowed types. In India, six-rowed barley predominates due to its higher yield and suitability as a feed crop, while two-rowed barley is preferred for malting purposes because of its uniform grain size. Malting barley is a premium commodity in the market, fetching higher prices as compared to food and feed types, owing to its specific quality attributes, such as low protein content, uniform kernel size, and suitability for brewing. The malt derived from barley is not only used for brewing but also as a nutritional supplement and an additive in various packaged foods. However, a consistent supply of malting barley is often limited by the lack of specialized malting varieties in India.

Barley Feed serves as a vital source of energy and protein for livestock like in dairy, beef, and poultry industry worldwide. Hulless barley, characterized by the absence of a fibrous hull, is higher in crude protein, starch, and *β*-glucans but lower in fiber, making it ideal for maximizing ruminal starch fermentation with minimal processing (2, 3). Conversely, hulled barley is more fibrous, requiring extensive processing like rolling or grinding to break the seed coat and improve microbial digestibility in the rumen (4, 5). Two-row barley typically has plumper kernels, higher starch content, and lower fiber than six-row types, contributing to better feed efficiency (4). However, kernel uniformity, test weight, and particle size distribution are critical for processing efficiency and digestion rates. Advances in barley breeding focus on traits like kernel uniformity and slower starch degradation to improve its suitability for various livestock. The strategic selection of barley types and processing levels ensures a balance between high energy release and minimal digestive disturbances, maximizing livestock productivity and health outcomes (6).

Barley for food purposes has a versatile role in traditional and modern culinary practices due to its nutritional profile and adaptability to various food applications. Pearled barley is widely utilized in traditional dishes across regions, such as soups, porridges, and flatbreads, barley products are also incorporated into breakfast cereals, soups, bakery flour blends, and baby foods (7). Incorporating barley flour into wheat-based foods significantly enhances their nutritional profile, particularly dietary fiber, *β*-glucan, and antioxidant activity, while imparting unique sensory attributes. Studies show that substituting 5–15% barley flour in wheat flour for producing bakery products maintains acceptable sensory quality, but impacts the loaf volume and other rheological properties (8). For noodles, 20–30% barley flour substitution retains desirable flavor and texture, with waxy barley types offering shorter cooking times and softer textures (9). Blending up to 30% hulless barley flour with wheat flour boosts *β*-glucan, phenolic content, and antioxidant activity (10). While biscuits and chapattis showed slight reduction in quality metrics, however it contributed to increase phenolic and β-glucan content, showcasing barley flour’s potential for functional wheat-based foods.

Several biochemical and structural traits influence the quality of barley suitable as malting types. Amylose, starch, and *β*-glucan content are key determinants of malt quality. Mixed-linkage (1 → 3; 1 → 4)-*β*-glucans, which constitute 75% of the endosperm and 26% of the aleurone cell walls, play a structural role in barley grains (11). However, elevated β-glucan levels are undesirable for malting, as they reduce malt extract yield and increase wort viscosity, leading to filtration challenges during brewing (12). Proteins, another critical component, influence malt quality in complex ways. Barley proteins include hordeins and glutelins, among others, but their exact roles in malt functionality remain incompletely understood (13). Starch, the primary carbohydrate in barley, undergoes structural changes during the malting process and its components, amylose and amylopectin, influence ferment ability and extractability of malt (14). Studies indicate that lower protein levels and reduced amylose content enhance fermentable sugar production during mashing, a critical factor for brewing (15).

The primary objective of barley breeding programs is to enhance both agronomic performance and quality traits. Agronomic traits such as yield, grain plumpness, and germination vigor significantly affect barley’s utility for malting. High germinability and superior seed vigor are essential for malting barley, as they determine the uniformity and efficiency of the malting process. Establishing correlations between agro-morphological descriptors (e.g., plant height, spike length, and grain weight) and biochemical traits like protein, starch, and *β*-glucan content is crucial for breeding programs aimed at developing malting-specific varieties. Breeding efforts should also address the trade-offs between traits desirable for malting and those advantageous for human consumption. For instance, high *β*-glucan and protein levels are beneficial for human health, contributing to dietary fiber and protein intake. Conversely, these traits can compromise malt quality by increasing wort viscosity and lowering malt extract yield (16).

The diverse applications of barley demand distinct biochemical and agronomic profiles, emphasizing the need for targeted breeding programs. Modern breeding strategies integrate molecular markers, agro-morphological traits, and biochemical parameters to address these varied requirements. Advances in molecular genetics and biochemistry have facilitated the identification of genes and pathways governing quality traits, enabling the development of superior barley varieties through marker-assisted selection and genomic breeding. Emerging technologies like genome-wide association studies (GWAS) and genomic selection (GS) hold significant potential for accelerating the breeding of high-performing barley, ensuring adaptability to diverse applications and environmental conditions. These holistic approaches are instrumental in addressing the complexities of barley improvement across food, feed, and malting industries.

Barley quality assessment is shaped by the intricate relationship between biochemical and agro-morphological traits. For example, *β*-glucan content has been shown to correlate with kernel plumpness, particularly in malting barley (17). However, most studies are confined to limited datasets comprising advanced breeding lines or established cultivars, which may restrict the discovery of novel traits. Expanding research to include diverse germplasm can provide valuable insights into previously unexplored agro-morphological and biochemical markers, enriching breeding programs and broadening the genetic diversity of barley. This study aimed to deepen the understanding of these interrelationships, offering critical insights into the factors that influence barley quality across its various applications.

India’s barley germplasm collection, housed in the National Gene Bank at the National Bureau of Plant Genetic Resources (NBPGR), comprises 8,183 accessions, including 1,509 exotic entries. This diverse repository holds immense potential for identifying trait-specific varieties. However, conducting comprehensive biochemical assessments for the entire collection is costly, time-consuming, error-prone, and environmentally unfriendly. NIR spectroscopy operates on the principle that near-infrared light induces twisting, bending, stretching, scissoring, or rocking alterations in molecular bonds with dipole movement, resulting in spectral variations that can be correlated with chemical composition. In this study, a reverse approach was applied, leveraging variation in NIR spectra to select a biochemically diverse subset. This technique facilitated efficient germplasm selection, enabling the identification of promising lines for food, feed, and malting applications.

## 2 Materials and methods

### 2.1 Sample collection and selection

A total of 5,733 hulled barley accessions conserved in the Long-Term Storage (LTS) of National Genebank at ICAR-National Bureau of Plant Genetic Resources (NBPGR) were used as base material for this study. All accessions were cultivated during the 2016–17 rabi season at the NBPGR experimental farm in Issapur, New Delhi, located at a latitude of 28°57’ N, longitude of 76°84′ E, and an altitude of 218 meters above sea level and stored in Medium Term Storage (MTS) at 4°C after harvesting (18). All Samples were homogenized in 2018 using a Foss cyclone mill and passed through a 1-mm sieve. The homogenized flour was scanned using an NIRSystems 6,500 scanning monochromator (FOSS, Hillerød, Denmark) in the range of 400–2,500 nm with a 2-nm interval in diffused reflectance mode. The spectral data were normalized using the standard Multiplicative Scatter Correction (MSC) method. Normalized spectra were analyzed through Hierarchical Cluster Analysis (HCA) using Ward’s method with squared Euclidean distance, followed by sub-clustering using the same approach (19). Representative samples were selected from cluster and sub-cluster centers and boundaries, forming a biochemically diverse subset of 136 accessions, which included six-row and two-row types as well as indigenous and exotic collections.

The selected set of 136 accessions, along with five check varieties, was evaluated in an Augmented Block Design at the ICAR-IARI campus, NBPGR New Area Experimental Farm, during the rabi seasons of 2019–20 and 2020–21. Data on agro-morphological and biochemical traits were recorded.

### 2.2 Biochemical estimations

All the procedures were carried out in triplicates and at room temperature (25°C).

#### 2.2.1 Estimation of starch

The total starch content was estimated as per using an assay kit (Megazyme, K-TSTA-100A, Wicklow, Ireland) AOAC Method 996.11 (20). The homogenized sample flour was extracted overnight with 80% ethanol, centrifuged at 13000 rpm for 10 min. The residue was used for estimation and involves use of Thermostable *α*-amylase to hydrolyze starch into branched and soluble/insoluble maltodextrin, further maltodextrin is hydrolyzed to D-glucose by amyloglucosidase. Glucose oxidase peroxidase (GOPOD) was used to oxidize the D-glucose into D-gluconate. The hydrogen peroxide liberated was estimated at 520 nm using Benchtop Lab Systems Spectrophotometer, after the development of pink color and the results were expressed as g/100 g.

#### 2.2.2 Estimation of amylose content

A modified iodometric method based on the amylose-iodine binding capacity (21) was used to estimate the AC in barley. The extraction process for AC involves incubation of homogenized sample with sodium hydroxide and absolute ethanol for 15 min over the water bath for 20 min. Gelatinized sample so obtained was diluted with distilled water to make up the volume to 50 mL and shaken vigorously. 500 μL sample aliquot were drawn from the dilutions in amber tubes. Further steps of process involving addition of 200 μL iodine solution (2 g iodine dissolved in potassium iodide, 20 g/L) under100 μL acetic acid conditions and a final dilution using distilled water and left for incubation, finally reading of absorbance of 620 nm were performed on Skalar San plus Analyzer. A standard calibration curve was also obtained using potato amylose (Sigma Aldrich) to validate the method, and the results were expressed as g/100 g.

#### 2.2.3 Estimation of protein

The total nitrogen content (%N) of rice was estimated by the AOAC 984.13 (20) with modified digestion method (22). The process involved pre-digestion of dried homogenized sample flour overnight with about 10 mL of ice-cold digestion mixture (sulfuric acid, selenium, hydrogen peroxide & lithium sulfate) and there by subjecting it to a temperature of 380°C for about 1 h to obtain a colorless solution, marking the complete digestion of the sample. Foss Tecator 2,300 Kjeltech Nitrogen Auto analyzer was calibrated with ammonium sulfate for 21% total nitrogen. The process followed by the auto analyzer was steam distillation of sample with 40% alkali (NaOH) to liberate ammonia which was further trapped with an indicator solution of 1% boric acid with bromocresol green/methyl red, accounting for the amount of total nitrogen present in the sample. The result so obtained was obtained %Nitrogen, and the total protein percentage was thus calculated by multiplying %N with a Jones conversion factor of 5.83.

#### 2.2.4 Estimation of total phenolic content

The total phenolic content was estimated using the Folin Ciocalteau reagent (FCR) spectroscopic method (23). The homogenized sample flour was extracted overnight for phenols with 80% ethanol, centrifuged at 13000 rpm for 10 min, and the supernatant was pooled. The sample aliquot of 500 μL from the supernatant was completely evaporated over a water bath at 100°C. Three mL of double distilled water was added to these test tubes and vigorously vortexed. Simultaneously blank was prepared by adding 3 mL double distilled water in separate tubes. For the preparation of standard, gallic acid (GA; 0.01, 0.02, 0.03, 0.04, and 0.05 mg) was added to a separate set of tubes and the volume was made to 3 mL. Subsequently, 500 μL of Folin–Ciocalteu reagent (FCR; equal part of FCR and water) was added to each of the three sets of test tubes (sample, blank and standards). Subsequently, 500 μL of Folin–Ciocalteu reagent (FCR; equal part of FCR and water) was added to each of the three sets of test tubes (sample, blank and standards). The test tubes were vortexed for 2 min, followed by the addition of 2 mL of 20% w/v, Na2CO3. After 1 h of incubation at room temperature, the absorbance of a dark blue-colored complex was measured at 650 nm using Benchtop Lab Systems Spectrophotometer. The FCR is a mixture of phosphomolybdate and phosphor tungstate, which gets reduced by phenols to form molybdenum blue under basic conditions. The measurement was compared to a calibration curve of Gallic acid, and the results were expressed as gallic acid equivalents (GAE) gram per hundred gram of sample (GAE g/100 g).

#### 2.2.5 *β*-Glucan content estimation

β-Glucan Content was estimated by Megazyme kit employed AOAC method 995.16 (20). The homogenized sample flour was extracted with ethanol and sodium phosphate in hot water bath for 5 min, followed by cooling to room temperature, thereafter 200 μL Lichenase enzyme was added and incubated at 40°C for 1 h. Subsequently volume was adjusted to 30 mL with distilled water. Lichenase breaks *β*-Glucan to *Β*-Gluco-oligosaccharides. The contents were vortexed and 100 μL of supernatant was drawn with one as reaction blank and two replicates for estimation. 100 μL of *β*-Glucosidase was added and incubated at 40°C for 15 min. Β-Gluco-oligosaccharides are further hydrolyzed to D-Glucose by β-Glucosidase. Glucose oxidase peroxidase (GOPOD) was added and incubated at 40°C for 20 min to hydrolyze D-glucose into D-gluconate. The hydrogen peroxide liberated was estimated at 510 nm using Benchtop Lab Systems Spectrophotometer, after the development of pink color and the results were expressed as g/100 g.

#### 2.2.6 Quality control

Quality control wase ensured by estimations of standard flours as recommended, carried out in duplicates with each lot of samples to ensure the reproducibility of the results and suitable standards and reagent blanks were used to ensure accuracy. ASFRM-Rice-2 from PT −8 obtained from INMU, Thailand, was used for method validation and check recovery of protein. At the same time, Total starch control kit (K-TSCK) flours, viz. wheat starch; high amylose maize starch were used for method validation of starch. Rice reference materials (BCR-465, 466, and 467) obtained from Sigma-Aldrich were tested for method standardization and validation of amylose estimation. Oat flour control powder included in the Megazyme assay kit was used as a standard for *β*-Glucan Content estimation.

### 2.3 Phenotyping of agro-morphological traits

The data were recorded on 10 agro-morphological traits of which 8 were in accordance with the descriptor list for barley (24),

**Table:** 

Trait	Code	Stage of observation	Descriptor status
days to 75% spike emergence	DSE	75% spikes emergence from sowing date	Plot basis
days to 80% maturity	DPM	80% spikes matured from sowing date	Plot basis
plant height	(PH; cm)	Recorded at maturity from ground level to spike top (excluding awns) of main tiller. Average of 3 random plants	Average in 3 replications
spike length	(SL; cm)	Length of spike (average of 3 random spikes per row)	Average of 3 replications
spikelet triplet groups per spike	(STG)	Number of spikelets (from triplets) per spike (average from 3 random spikes) post-harvest.	Average of 3 replications
grain number per spike	(GNS)	Grain number from 3 random spikes post-harvest	Average of 3 replications
Hundred-grain weight	(HGW; g)	Post harvest	Average of 3 replications
Grain yield /per meter row	(GY; g)	Total grain yield	Average of 3 replications

Remaining two descriptors were recorded as under.

Grain filling time (GFT): Calculated as difference between days to 80% maturity and days to 75% spike emergence.

GFT = DPM-DSE.

Grain width to length ratio (GWL): Seeds from three random plants were scanned on flatbed scanner and TIFF image was evaluated using Matlab based software for measurement of plant parts v1.3 developed by ICAR-Central Institute for Agriculture Engineering, Bhopal. This was recorded as a representative of the plumpness of grains.

### 2.4 Statistical analysis

Traits relating to Agro morphological descriptors were considered as a single group while traits relating to biochemical descriptors were considered as biochemical group. Mantel test was performed between these two groups to ascertain any significant interrelation between the traits. Pearson’s Correlation analysis was conducted to determine the relationships between the individual agro-morphological trait and the biochemical traits. Each of the biochemical and agro morphological traits was considered as independent trait for drawing the correlation coefficients. Correlation coefficients generally show linear relationships among independent characteristics. All the statistical analysis was done using R 4.2 package. Factominer was employed for PCA analysis with center and scale parameters as true. Hierarchical clustering was performed using Ward D2 method with Euclidean distance.

## 3 Results and discussion

This study identified a subset of barley accessions exhibiting complete range of diversity for biochemical and agro-morphological characteristics. This diversity provides a valuable resource for advancing barley breeding programs aimed at improving both biochemical and agronomic traits. By combining diverse biochemical profiles with agro-morphological performance, these accessions aids in understanding the interrelations between traits and provide a foundation for developing high-performing barley varieties.

Importantly, breeders are more interested in studying biochemical traits in interaction with agronomic traits rather than in isolation. An integrative approach is essential for effective selection and breeding strategies. This approach enables the identification of genotypes that excel in both productivity and nutritional quality. A critical tool for understanding these interactions is the calculation of phenotypic correlation coefficients, which quantify the relationships between nutritional parameters/biochemical traits (e.g., protein content) and agro-morphological traits (e.g., plant height, grain yield).

By exploring these correlations, breeders gain insights into how biochemical traits align with or influence desirable agronomic characteristics, facilitating informed decision-making in breeding programs. This approach ensures that the development of new barley varieties addresses the complex interplay of traits required for both agronomic performance and nutritional enhancement, paving the way for sustainable crop improvement efforts.

### 3.1 Biochemical profiling

All parameters, except amylose, showed significant variability, with the greatest variation observed in starch content, followed by *β*-glucan. The study identified high-protein lines with consistently above-average protein levels. Additionally, several lines with advantageous traits for malting were identified.

#### 3.1.1 Starch content

Starch is a major component of barley endosperm and is most important constituent from barley utilization perspective as it is the basic substrate for various amylases and therefore is important in syrup, feed and malting, and brewing industries (25, 26). The visco-amylo-graphy of barley starches provides information on their pasting characteristics and textural changes during cooking and cooling cycles. Physiochemical factors of starches of cereals crops determine their end use (27). Viscoamylographs have been exploited in simulating commercial processing conditions in brewhouse, assessing the effects of pH and temperature on starch gelatinization, and identifying significant changes in viscosity associated with proteolytic and saccharification activity (28). The Starch content in Barley generally is 51–64% (25) of the total composition and also an important trait for food, feed as well as malting perspective. Our dataset showed a wide range of starch content in range of 32.4 to 60.3%/100 g of flour which was at par with 45.7 to 66.4% (26) (Table 1). Lowest starch content was exhibited by EC0578430 with 32.45% and highest starch content was exhibited by EC0329008.The average starch content for our data set was 49.8% per100 g.

**Table 1:** Descriptive statistics of the five biochemical parameters of barley.

Parameter	Range (%)	Mean ± SD
(AC) Amylose (g/100 g)	13.3–19.3	16.1 ± 1.08
(TPC)Phenol (GAE g/100 g)	0.073–0.67	0.30 ± 0.11
(PC) Protein (g/100 g)	8.6–17.4	12.5 ± 1.84
(SC) Starch (g/100 g)	32.4–60.3	49.8 ± 4.5
(β-gC) β-Glucan(g/100 g)	1.31–6.06	3.56 ± 1.08

SD, standard deviation.

Starch is an important constituent as differential starch digestion rate is a critical factor when barley is used as food for humans or feed for animals. For human consumption, a slower digestion rate is beneficial, as it may help manage conditions such as obesity, diabetes, and colorectal cancer. Conversely, a faster digestion rate is desirable in animal feed to promote rapid weight gain in growing animals and provide high energy availability during food production. Medium and low starch accessions identified in this study may be further assessed for digestion rates.

Higher starch content utilization has been associated with exploitation for malting brewing industry.

#### 3.1.2 Amylose content

Amylose is an important constituent of starch and affects its physiochemical properties. High amylase content is undesirable for brewing industry as interferes in starch hydrolysis during mashing (29) Amylose content for normal type barley varieties ranges from 5 to 35% (30) of total composition. Less than 5% are considered waxy type and greater than 35% are considered high amylose type (30). In our study the range for the set was found to be narrow 13.3 to 19.3%. The lowest amylose content of 13.3% was found to be of EC0578330, highest amylose content 19.3% was found to be of EC0492138. Though none of the accession identified could be scored for as waxy type or high amylose type, this subset can still be utilized for developing breeding lines for feed barley.

#### 3.1.3 Protein content

Malting varieties are developed for a specific grain composition wherein maximum prescribed limit for protein concentration of 12.0% (29). Barley endosperm protein is rich in prolamin storage proteins which has moderate nutritional quality, however, it can be improved to provide high quality protein through mutant lines and breeding programs (31). Protein content in barley is generally from 8.02 to 13.5% (30). Our set had a wide range of 8.6 to 17.4% with a mean of 12.5% comparable to as reported by (32) (Table 1). Low Protein content is a desirable trait from brewing perspective, however high protein content is favored for food and feed quality. IC0446085 exhibited lowest protein content 8.6% and highest 17.4% was of EC955621. Low protein is desirable trait for malting type barley have been generally associated with plump grain and lower protein content.

#### 3.1.4 Total phenol content

Polyphenolic compounds are a constituent of barley grains, malt and distilled as well as un-distilled spirits produced from these malts. Polyphenols play a crucial role in providing flavors to the beers and stability during storage (33). However, Polyphenols have also been reported to form colloids with proteins and cause haze, which is an undesirable trait for consumers (34). Therefore, a controlled amount of phenolics are desirable in the malt/brewing related Barley varieties. Polyphenols being a powerful source of antioxidants are also desirable for their health benefits.

However, there are not many accounts of Total phenol content from broad genetic base material in barley. TPC content has been reported as 0.217 to 0.256% (35). Our set showed a wider range of 0.073 to 0.67%. EC0578951 had the lowest value 0.073% and EC955621 had highest value 0.67% (Table 1).

#### 3.1.5 *β*-Glucan content

β-Glucan has emerged as a holy grail of health benefits. Various studies have revealed it to be linked with reduction in the risks of chronic health problems, such as those associated with cardiovascular diseases because of reduction in blood cholesterol and those associated with diabetes as it regulates blood glucose levels (36, 37). It is also associated with weight loss and regulating blood pressure (38). *β*-Glucan content in our study has been broadly represented in the range of 1.31 to 6.06% (Table 1). Our values were comparable to as reported by (39). IC0026547 had the lowest value and EC955451 had the highest value.

### 3.2 Identified promising accessions

In addition to the accessions, we already identified, we found some promising ones that could be used to develop new barley varieties for food, animal feed, and brewing. For brewing, low protein content is important because high protein can make beer cloudy, which is not preferred. Accession EC0329008, with a very low protein content of 8.8% and high starch content of 60.3%, is ideal for brewing malts. Another good candidate is IC0532985, which has 8.22% protein and 57% starch.

*β*-Glucan is another trait that should be low for good brewing and feed quality. We identified EC955656 and EC0492138, which have low *β*-glucan levels (1.6 and 1.5%, respectively), low protein (9.4 and 9.5%), and high starch (54 and 59.7%), making them suitable for brewing. High content of protein and β-glucan is desirable for human food and animal feed, accession EC955451, with high β-glucan (6.06%) and high protein (15.6%), and is a good candidate.

### 3.3 Agro-morphological traits

Agro-morphological descriptors of Indian Barley Germplasm have been studied and reported in detail (18). The study has reported a wide range for each descriptor. Assessment and correlation of agro-morphological traits with biochemical descriptors also provided insights in respect of the performance of NIRS based approach vis a vis agro morphological diversity matrix of the selected biochemically diverse representative set.

This biochemically diverse set had nearly equal representations from the 6 row and 2 row type hulled barley. All the accessions showed wide variability for agro morphological traits as given in Table 2. Around 60% of accessions were intermediate in spike emergence (80 to 95 days) and maturity (125–140 days). Only one accession IC0445972 was early heading type (<75 days). Similarly, only one accession IC0355876 was early maturity type (<120 days). The mean range of 75% spike emergence ranged from 74 to 124 Days, while the means DPM ranged from 119 to 147 days. Barley being lodging prone crop species low PH is a desirable trait. Average value for PH was 116 cm which was at par with as reported in (18). However, for this biochemically diverse data set, PH lied in the intermediate range, i.e., 82 to 165 cm and none of the accession was recorded to be of dwarf type (<75 cm).

**Table 2:** Descriptive statistics of agro-morphological traits of barley.

Parameter	Range	Mean ± SD
DSE(days)	74–124	94.5 ± 10
DPM(days)	119–147	131 ± 7
GFT(days)	13–67	37 ± 9
PH(cm)	82–165	116 ± 14
SL(cm)	5–11	8 ± 1
STG(number)	13–35	24 ± 5
GNS(number)	15–71	37 ± 15
HGW(g)	2–6.7	4.3 ± 0.8
GY(g)	13.7–236.31	100.9 ± 43.4
GWL(ratio)	0.23–0.47	0.32 ± 0.041

SD, standard deviation.

Average GY for 2-row accessions (109.01) was slightly higher than the 6 row (95.7) accessions. Ranges with average for SL, STG and HGW as provided in Table 2 did not show any such trend for 2 row and 6 row barley types.

An estimate of GFT ranged from 13 days to 67 days. GFT, i.e., Grain filling time has been well researched in recent times as reported in barley (40) and in maize (41). Grain filling time is affected by temperature and other climatic conditions and therefore in view of rising global temperatures, is an important trait of assessment. Specific studies designed to assess climatic conditions, grain filling time and its impact on other quality and agronomic traits may help in future breeding programs.

### 3.4 Statistical analysis

#### 3.4.1 Mantle test

The Mantel test, introduced in 1967, is commonly used to assess the association between two matrices and has been extensively applied to examine relationships such as geographic distance and genetic divergence (42). In this study, Mantel test was utilized to explore potential relationships between agro-morphological traits and biochemical traits, treating these two groups as separate matrices for the same set of samples.

To evaluate the associations between these distance matrices, the Mantel test was conducted using Pearson’s product–moment correlation, with the *p*-value determined from the distribution of r(AB) derived through 10,000 permutations. The Mantel statistic indicated a weak and non-significant correlation between the two trait groups, with *r* = 0.0267 and *p* = 0.07.

#### 3.4.2 Pearson’s correlation

Pearson’s correlation coefficient was drawn between biochemical and agro-morphological traits to further understand their inter-relationship as provided in Table 3.

**Table 3:** Pearson’s correlation coefficients drawn between all the traits including 5 biochemical and 10 agro morphological traits.

	TPC	PC	BgC	SC	AC	DSE	DPM	GFT	PH	SL	STG	GNS	HGW	GY
PC	0.333***	—												
BgC	−0.029	0.269**	—											
SC	−0.079	−0.409***	0.089	—										
AC	−0.276**	−0.398***	−0.135	0.48***	—									
DSE	−0.003	0.036	0.026	−0.007	−0.087	—								
DPM	−0.029	−0.088	0.022	0.106	−0.058	0.552***	—							
GFT	−0.019	−0.112	−0.014	0.092	0.057	−0.753***	0.133	—						
PH	−0.147	0.113	0.168	−0.021	−0.071	−0.023	0.251**	0.225**	—					
SL	−0.118	0.003	−0.062	−0.024	−0.013	0.002	0.079	0.06	0.18*	—				
STG	−0.093	0.013	0.087	−0.008	0.068	0.116	−0.111	−0.225**	0.107	0.475***	—			
GNS	0.012	0.036	0.012	−0.031	−0.074	−0.111	0.171*	0.267**	0.115	0	−0.153	—		
HGW	−0.09	−0.003	−0.002	0.052	0.023	−0.131	−0.105	0.072	0.155	0.253**	0.307***	−0.305***	—	
GY	−0.014	0.144	0.04	−0.037	0.048	−0.235**	0.018	0.294***	0.299***	−0.021	0.037	0.009	0.311***	—
GWL	−0.235**	−0.001	0.257**	0.203*	0.111	0.125	0.115	−0.058	0.121	−0.133	−0.095	0.083	−0.186*	−0.022

TPC, Total Phenol content; PC, protein content; BgC, β-glucan content; SC, starch content; AC, Amylose content; DSE, Days to 75%spike emergence; DPM, Days to physiological maturity; GFT, Grain filling time; PH, Plant Height; SL, Spike length; STG, Spikes per triplet; GNS, Gain number per spike; HGW, Hundred Grain weight; Yield, yield /met row(GY); GWL, Grain Width length ratio.

Highly significant positive correlation was found between TPC and PC (*r* = 0.33) as also reported in (43). The reason for these repeated similar finding may be attributed to phenolic group being an excellent hydrogen donor that forms hydrogen bonds with hydroxyl group of protein and therefore develops an affinity for the same, besides there a number of mechanisms are suggested by (44) for protein and phenol interaction. Highly significant negative correlations were observed between SC and PC(*r* = −0.4) as reported by (29) and AC and PC (*r* = −0.41) similar to as reported by (45).SC and PC negative correlation may be resultant of photosynthates being diverted to protein accumulation in certain cultivars.

Significant negative correlations were observed between AC and TPC (*r* = −0.27) a possible reason for same may be due to V type Amylose inclusion helical complex as suggested in (46). Phenolic compounds are tightly complexed inside the cavity of amylose helices and therefore become resistant to washing even repeated washing with 50% v/v ethanol solution which is extraction step for Folin Ciocalteu method of phenol estimation as mentioned above.

*β*-gC was found to be significantly positively correlated (*r* = 0.2) with PC, as suggested by (46) which reviews different authors and mentions that that β-glucan deposition was associated with protein accumulation in oats. AC being a component of Starch, highly significant positive correlation (*r* = 0.48) between them is observed.

Some highly significant correlations were observed for agro-morphological traits as well. DSE was found to have highly significant positive correlation with DPM (*r* = 0.55) which may be obvious as spikes experiencing later will result in spikes reaching physiological maturity later to the average no of days recorded. DSE also showed negative correlation (*r* = −0.75) with GFT. Late spike emergence led to fewer days for grain filling as physiological maturity which is dependent on temperature rise is achieved early in northern Indian plains, therefore late spike emergence meant short GFT. Similar result was reported by (47). GY showed highly significant positive correlation with GFT (*r* = 0.29) and PH (*r* = 0.29) (48). reported a negative relationship between PH and GY in wheat. Besides, above significant correlations were observed between GFT showing significant positive correlation with PH (*r* = 0.22), GNS (*r* = 0.26) and significant negative with STG (*r* = 0.22).PH was significantly positively correlated with DPM (*r* = 0.2) and HGW with SL (0.25).

No significant correlations were observed between HGW and any of the biochemical traits. The husk content in the homogenized hulled barley sample may account for this observation, as the biochemical constituents analyzed are primarily part of the kernel, and the husk-to-kernel weight ratio is highly variable. Previous reports have indicated that the proportion of hull in selected breeding lines of hulled barley ranges from 10.2 to 20.7% (49). However, the grain width-to-length ratio (GWL) exhibited a significant positive correlation with *β*-glucan content (β-gC, *r* = 0.257) and a significant negative correlation with total phenolic content (TPC, *r* = −0.235), consistent with findings by (17). Additionally, GWL showed a weak but significant negative correlation with HGW (*r* = −0.186), suggesting that bold grains typically have a lower husk proportion compared to slender grains. High grain yield and plump grains are preferred traits in quality breeding due to their impact on total malt yield per batch. These findings indicate that GWL may be a more effective grain morphological trait than HGW for selection in quality breeding programs for hulled barley.

In our study Biochemical traits were found to have strong correlation with other biochemical traits, likewise the agro-morphological traits were also found to be strongly linked to other agro morphological traits. These results are in solidarity with the Mantle test results as discussed above.

Positive correlations between SC and AC are obvious and well reported. Positive correlations between PC and *β*-gC and PC and TPC suggest sergeant selection on basis of PC may affect selection of these two traits accordingly. High β-gC and PC is desirable for human food consumption but is not a desirable trait for brewing and feed quality. Specific cultivar development for specific needs such as high β-glucan, high protein being favorable for human food, similarly low β-glucan and high protein content being good for feed consumption and low protein, low β-glucan being desirable for brewing has been suggested in (17). These correlation studies provide insights into selection of cultivars and development of material during breeding programs.

#### 3.4.3 Principal component analysis

PCA being a dimension reduction method reduces the possibly correlated variables into a set of values of linearly uncorrelated variables called principal components. The first principal component captures the greatest amount of the variance in the data. The second principal component explains the greatest amount of the variance in the data that is not captured by the first principal component and so on.

**Figure 1:**
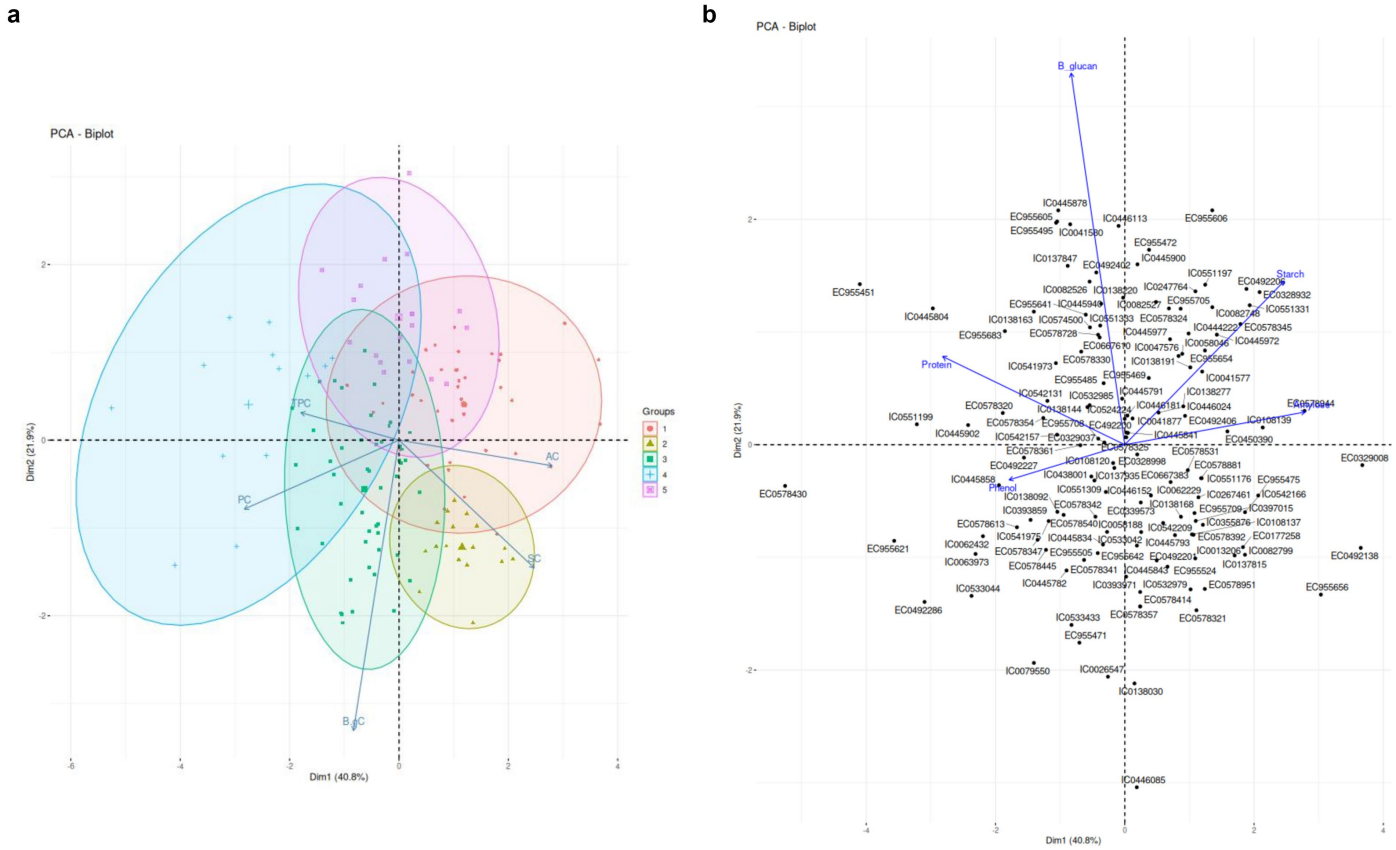
**(A)** PCA Biplots based on Biochemical Traits. **(B)** Accession aligned with PC component loading based on biochemical traits.

Figure 1A pictographically represents the loadings of the various components. Two PCs were identified with eigenvalue>1 which contributed the maximum in data variability. The PC1 captured variability caused by four out of five biochemical parameters and contributed to 41% variance of the data. PC2 was attributed to *β*-gC and contributed to 22% variance in the dataset. The maximum loading was observed for Protein in PC1 (0.745) followed by other biochemical traits. PC2 was clearly attributed to β-gC (0.930) with slight contribution from SC. The PC2 attributing to highest β-Glucan loading (0.935) validates no significant correlations between *β*-Glucan and other biochemical traits which is also evident in Figure 1.

Figure 1B details accessions closely aligned with each biochemical trait. Such as EC0578944, EC0108139, EC0450390 were found to be better associated with AC, EC955654, EC0578345, EC0328932, IC0551331, IC00446024 were better associated with SC. IC0542131, IC0138144, EC977708 and EC0329037 were found to be better associated with PC. TPC was found to be better associated with IC0108120, IC0445858 and EC0492227. β-gC was found to be better associated with EC0492402, IC044590, IC0551333, EC955469 and IC0445791.

The identified accessions possess traits or characteristics that hold potential use in targeted breeding programs. These genetic resources can be systematically utilized through selection strategies to isolate desirable traits. Alternatively, hybridization approaches can be employed to combine complementary traits from different accessions, generating new genotypes which may be apt for differential uses in food, feed and malting.

**Figure 2:**
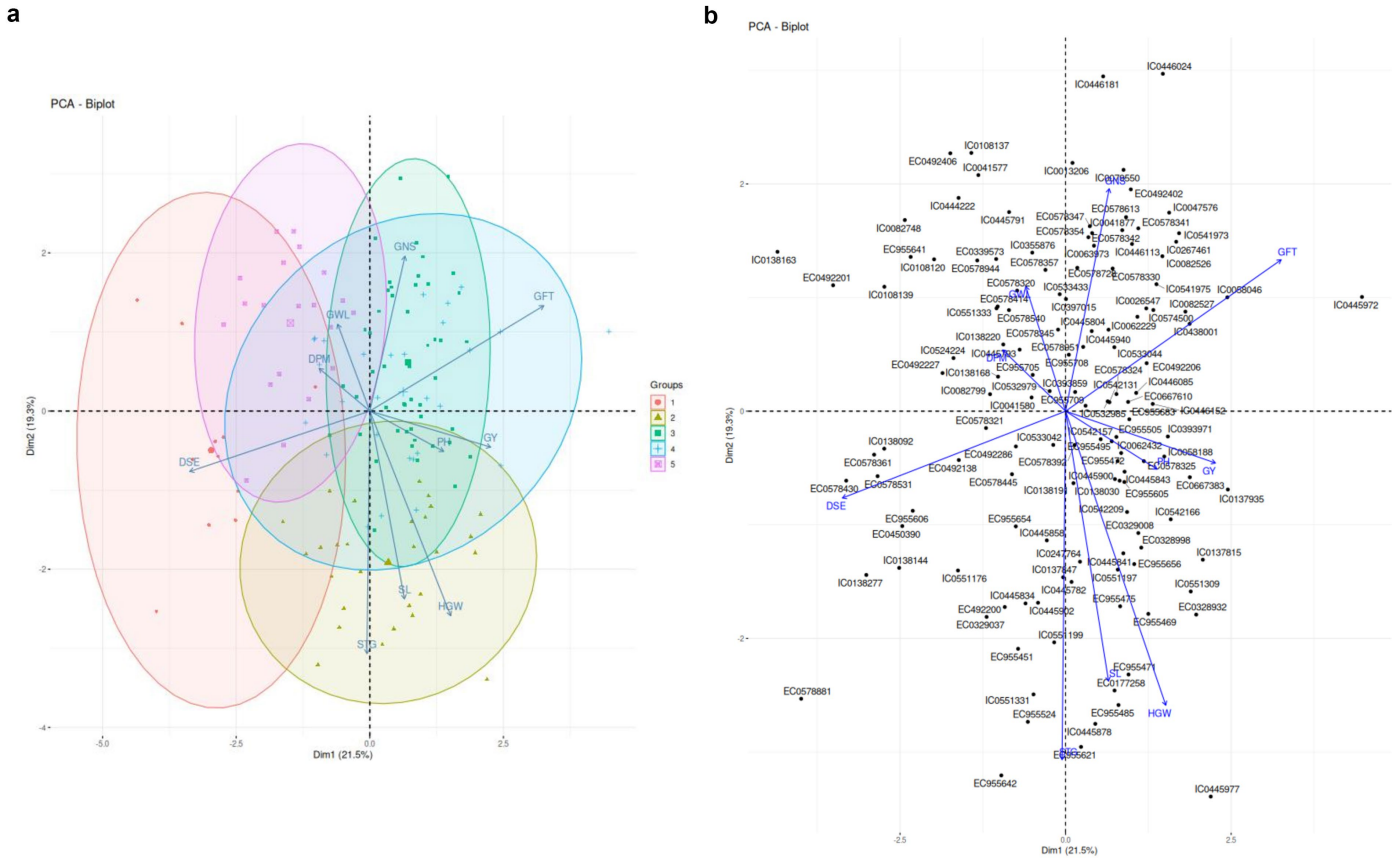
**(A)** PCA Biplots based on Agromorphological Traits. **(B)** Accessions aligned with PC component loadings based on Agro-morphological traits.

Figure 2A depicts the principal component loadings of agro-morphological traits of barley dataset. Three major components PC1, PC2, PC3 with eigenvalue >1 was identified which contributed to 62.6% of variance in this dataset.

PC 1 captured variance caused by 5 out of 10 parameters, i.e., DSE, GFT, GY, HGW and PH and attributed to 21.5% variance as shown in Figure 2B. DSE has been major contributor followed by GFT and so on. PC2 attributed to 19.3% variance with STG being major contributor.PC3 contributed to 16.5% variance and can be majorly attributed to DPM followed by PH.

PCA plot also revealed certain accessions closely related with agro-morphological traits. STG, SL, HGW, PH and Yield aligned closely which indicates possible correlations among these traits. EC 955606, EC0578321, IC0041580 better associated with DSE. EC955709, IC0138168 aligned with DPM. EC0578324, EC955683, EC0492206 better associated with Grain filling time. EC 955505, EC955472 and IC0058188 better associated with yield.PH was found to be better associated with IC0542157EC 9,555,495, EC955605. HGW better associated with IC0551197. SL better associated with EC0177258, EC955471, STG better associated with IC0445782, IC0137847 and IC0551331. GNS associated with EC0518951, IC0533044 and IC0542131.

As the Principal Component Analysis (PCA) for both biochemical and agro-morphological traits appeared to lack clear separation or distinct groupings. This diffusion in cluster formation indicates an overlap in trait variation, suggesting that the data does not readily lend itself to categorizing subsets with specific, actionable characteristics. Consequently, the analysis fails to provide guidance for the targeted utilization of these traits, such as breeding programs or resource allocation. This outcome highlights prompted us to further refine the study and apply Hierarchical clustering.

#### 3.4.4 Hierarchical cluster analysis

**Figure 3:**
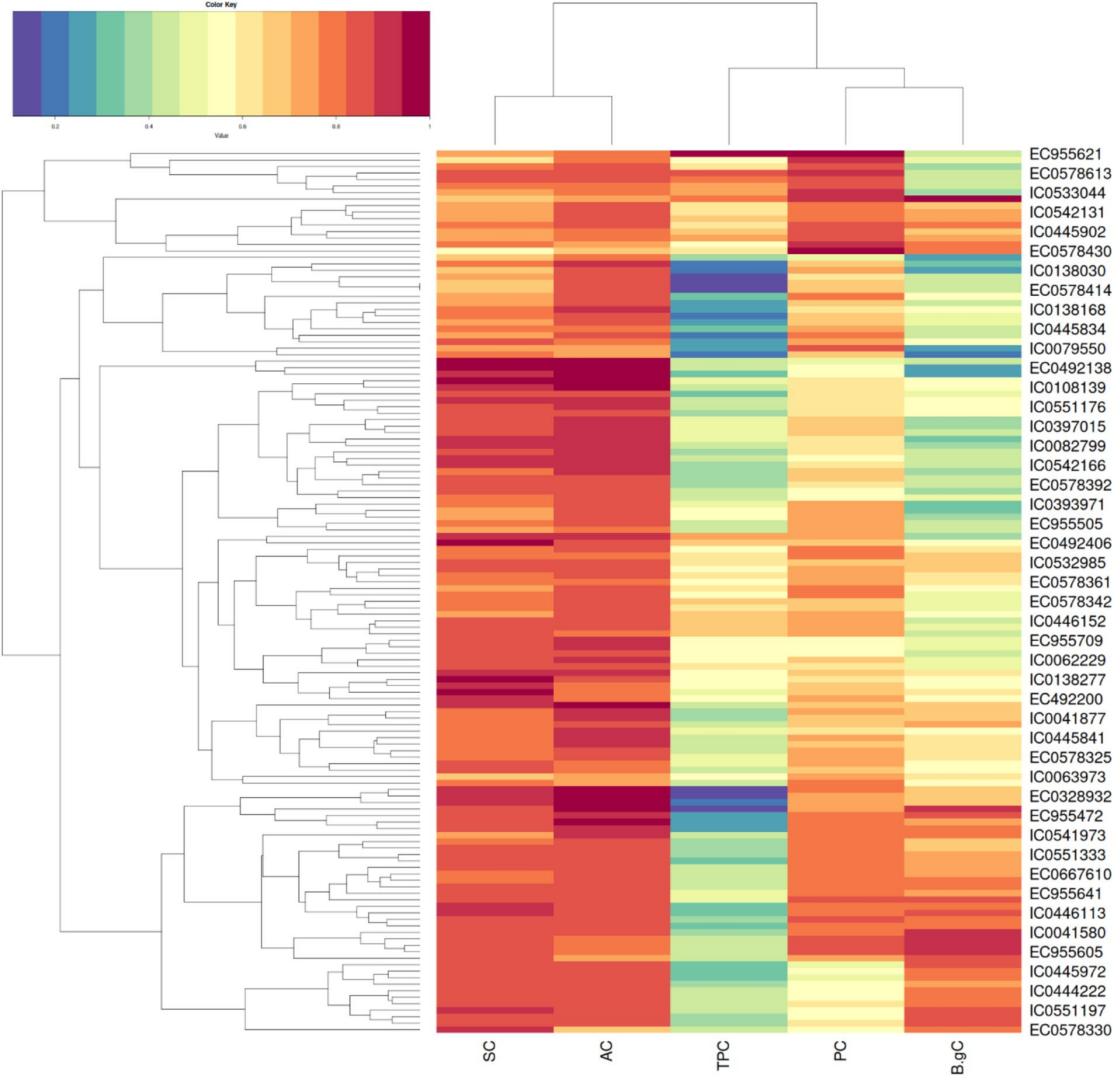
Heat map showing the clusters distribution on basis of biochemical traits.

**Figure 4:**
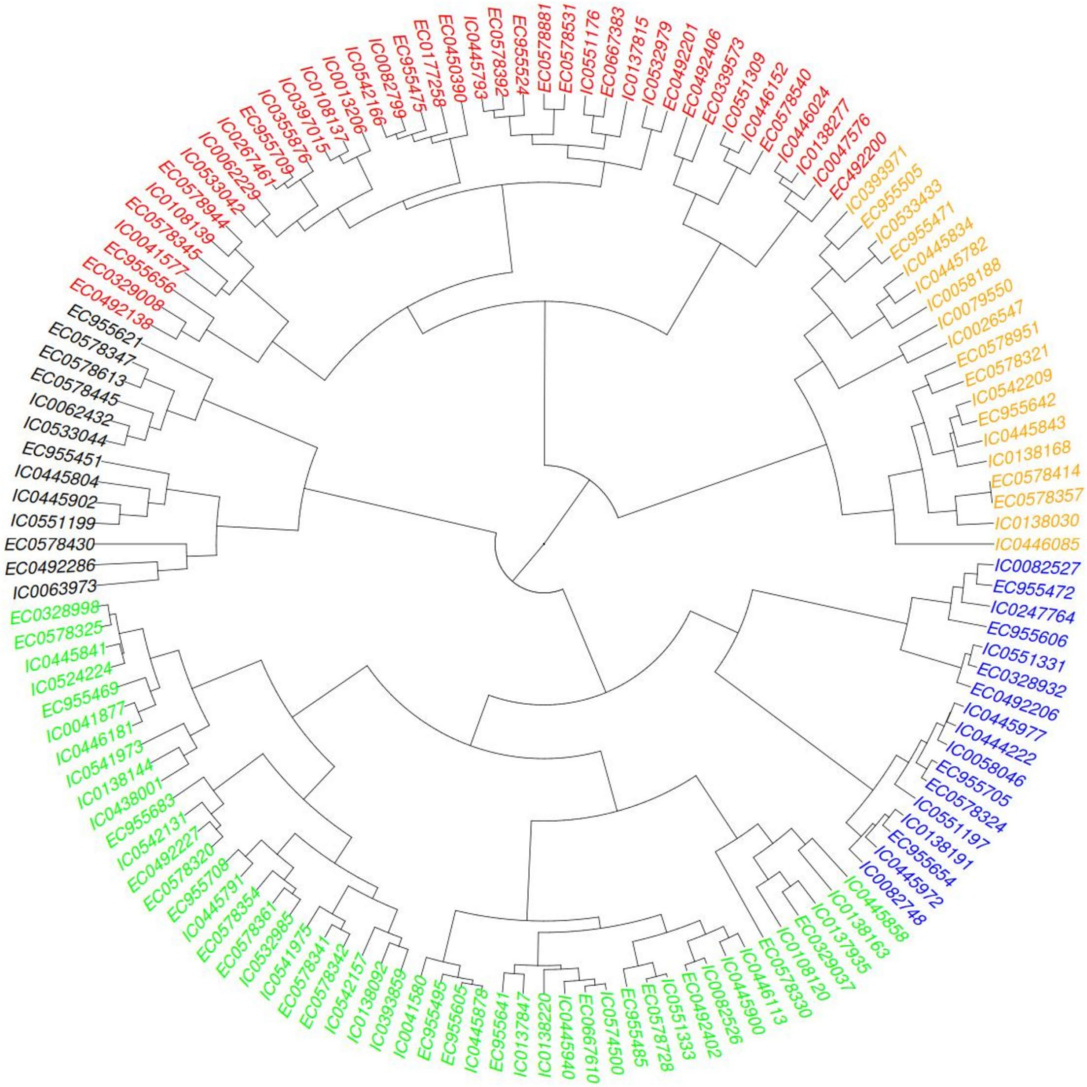
Clusters based on biochemical traits.

Hierarchical clustering as depicted in Figure 3 on basis of biochemical traits was able to reveal 5 major distinct clusters. Euclidean square distance of 5 was used as a distance linkage metric between groups. Figure 4 depicts the distribution of hierarchical clusters based on biochemical traits. Table 4 provides an insight into the agro-morphological traits associated with these clusters. The average values of the agro-morphological traits of these clusters revealed some patterns.

**Table 4:** Means of agro-morpho traits of individual clusters (5) obtained from hierarchical cluster analysis on basis of biochemical traits.

Cluster	Cases per cluster	DSE	DPM	GFT	PH	SL	STG	GNS	HGW	GY	GWL
I	13	96.2	130	33.8	114.9	8.6	25.1	41.8	4.4	99.9	0.29
II	19	92.3	130.4	38	117.7	8.8	25.1	36.6	4.3	106.0	0.31
III	48	93.9	132.1	38.2	118.1	8.3	23.5	37.7	4.2	103.5	0.33
IV	39	96.3	133.2	36.8	112.1	8.5	24.1	35.8	4.2	89	0.32
V	17	92.8	131.7	38.8	121.9	8.7	24.4	38.5	4.5	116.3	0.31

As provided in Figure 4 Cluster I had 13 members characterized with high TPC (0.35 to 0.67%), high PC (12.59 to 17.45%) and moderate to low SC (32.48 to 50.18%)and AC (13.38 to 16.36%) with *β*-gC (2.18 to 6.06%).

Cluster II exhibited 19 members characterized with low TPC (0.07 to 0.38%,), moderate to low β-gC (1.31 to 3.19%), PC (8.67 to 14.67%), SC (40.56 to 49.7%)and AC (14.48 to 17.21%).

Cluster III exhibited 48 members characterized moderate to high *β*-gC (2.83 to 5.62%), TPC (0.16 to 0.47%), PC (10.11 to 14.99%), SC (42.62 to 54.91%), AC (13.27 to 17.34%).

Cluster IV exhibited 39 members characterized moderate to high SC (47.77 to 60.3%) and AC (15.61 to 19.32%).TPC (0.21 to 0.49), PC (8.82 to 13.26%), β-gC (1.58 to 4.56%).

Cluster V exhibited 17 members with low to moderate TPC and low PC. TPC ranging between 0.08 to 0.29 GAE g/100 g, PC ranging from 9.12 to 13.82, β-gC ranging from 3.97 to 5.41% per 100 gm, SC ranging between 50.22 to 55.91% per 100 gm, AC ranging from 16.44 to 18.68% per 100gm.

Cluster I is formed by accessions rich in protein and phenols and moderate to high β-glucan, offers significant nutritional benefits, including antioxidant properties and enhanced dietary fiber intake. These attributes make it ideal for developing functional foods, enhancing nutritional value in cereals, and potential therapeutic uses (50). Moreover, barley protein is superior in essential amino acid profile compared to many cereals, particularly rich in lysine, enhancing its nutritional quality and utility in protein-enriched diets (51). The agro-morphological traits of this cluster showed trending toward early spike emergence combined with above average GFT indicating toward late maturing types which may be suitable for suitability for regions needing intermediate growing seasons.

Cluster II is characterized by accessions with low to very low phenol content, moderate to high starch, moderate amylose and *β*-glucan, and moderate protein levels. Low phenol and low β-glucan levels are advantageous for brewing and malting, as they improve beer clarity and reduce viscosity issues during processing (52, 53). The amylose content of about 25% is beneficial for brewing due to its impact on starch gelatinization and enzymatic breakdown, which are crucial for malt quality (54). The cluster members showed concentration of late maturing cultivars with below average DSE and higher than average DPM suggesting suitability for winter type environments also below average PH which indicates reduced lodging.

Cluster III has accessions have high *β*-glucan and phenol levels, moderately high protein and starch. These characteristics are beneficial for human food and animal feed applications. These accessions are suitable for products that require nutritional balance and functional properties like viscosity and texture (54, 55). Additionally, barley beta-glucan enhances gut health by promoting beneficial microbiota growth and fermentation, leading to improved gut barrier function and immunity (56). This cluster had below average GFT which indicated toward early maturation type cultivars which is a promising trait from agronomic perspective.

Cluster IV comprises accessions characterized by low *β*-glucan, high phenol content, low protein, and high starch with optimal amylose levels for gelatinization and enzymatic starch breakdown. This combination, particularly the elevated polyphenols and low *β*-glucan, is advantageous for producing high-quality barley malt-based distilled spirits, where polyphenols not only act as antioxidant but also contributes to flavor and aging characteristics in whiskey, enhancing its sensory attributes (57–59). Members exhibited below average DSE and DPM suggesting early maturity therefore suitable for winter type cultivation. Further above average GY combined with biochemical traits makes it promising for exploiting in yield related crop improvement programs.

Accessions in Cluster V, marked by low phenol, low protein, and high starch with elevated *β*-glucan, are nutritionally significant for their high soluble fiber content, beneficial for heart health and glycemic control. In the food industry, these traits are advantageous for developing high-fiber products and functional foods (56, 60). Below average PH for this cluster suggested suitability toward lodging resistance, however below average GY indicated requirement for improvement of yield contributing traits.

The clustering confirms that starch and *β*-glucan are major contributors to the variance observed in this dataset. Accessions with lower β-glucan levels tend to cluster with low phenol and moderate to high amylose and starch, suggesting divergent selection pressures for β-glucan and amylose during breeding cycles (17). Cluster I and III members are more suitable for human food and animal feed due to high protein and moderate β-Glucan content (31). High protein content is undesirable for brewing purposes as it has been reported to cause haze and interfere with foam stability (61). However, this group may be exploited for utilization in food products such as breads and “sattu” a traditional Indian food product prepared by roasting hulled barley followed by grinding it to a fine powder and sieving. These findings provide a comprehensive understanding of the biochemical traits and potential applications of the accessions studied, guiding their use in health food and industrial contexts.

**Figure 5:**
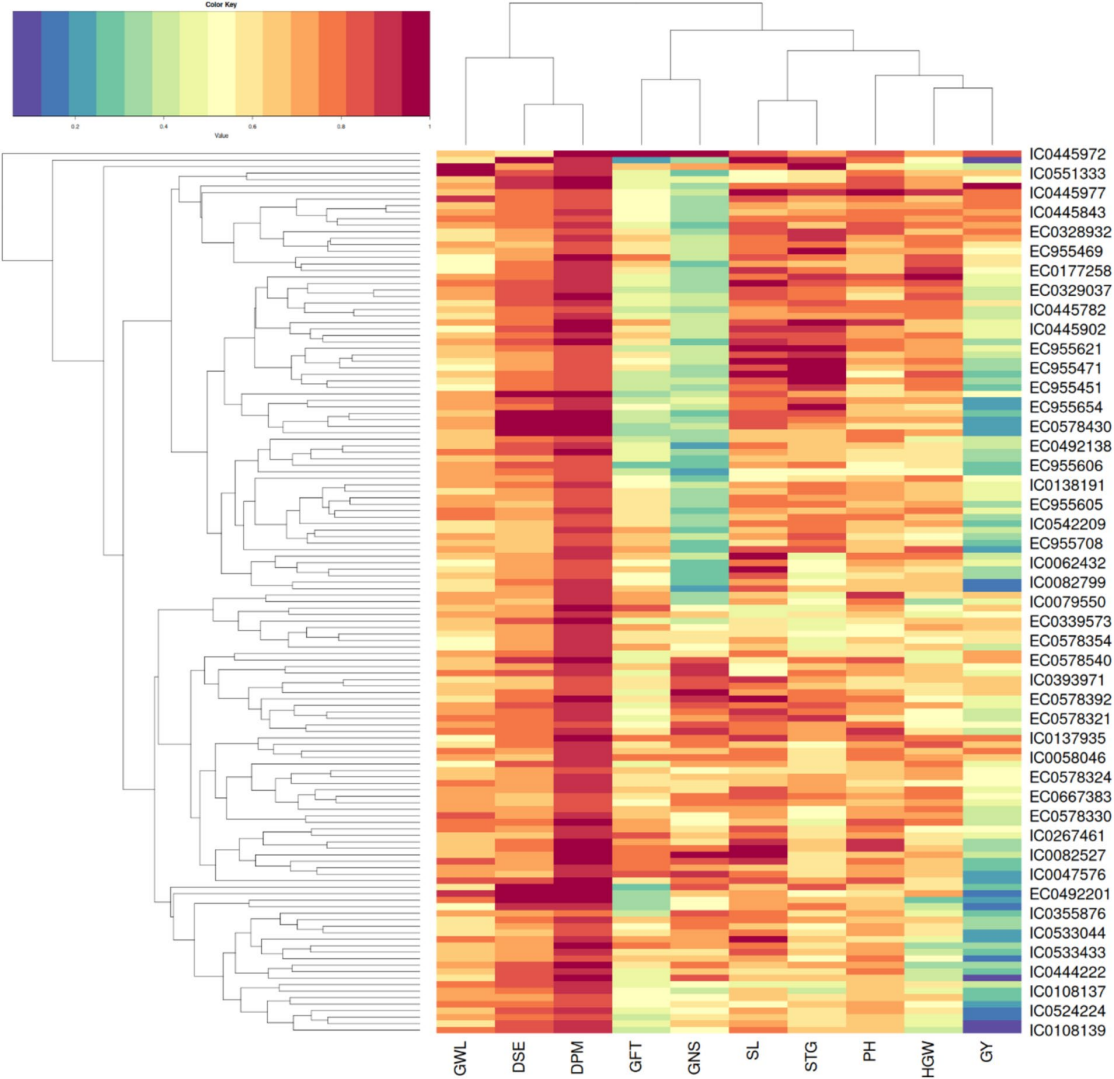
Heat map showing the clusters distribution on basis of agro-morphological traits.

**Figure 6:**
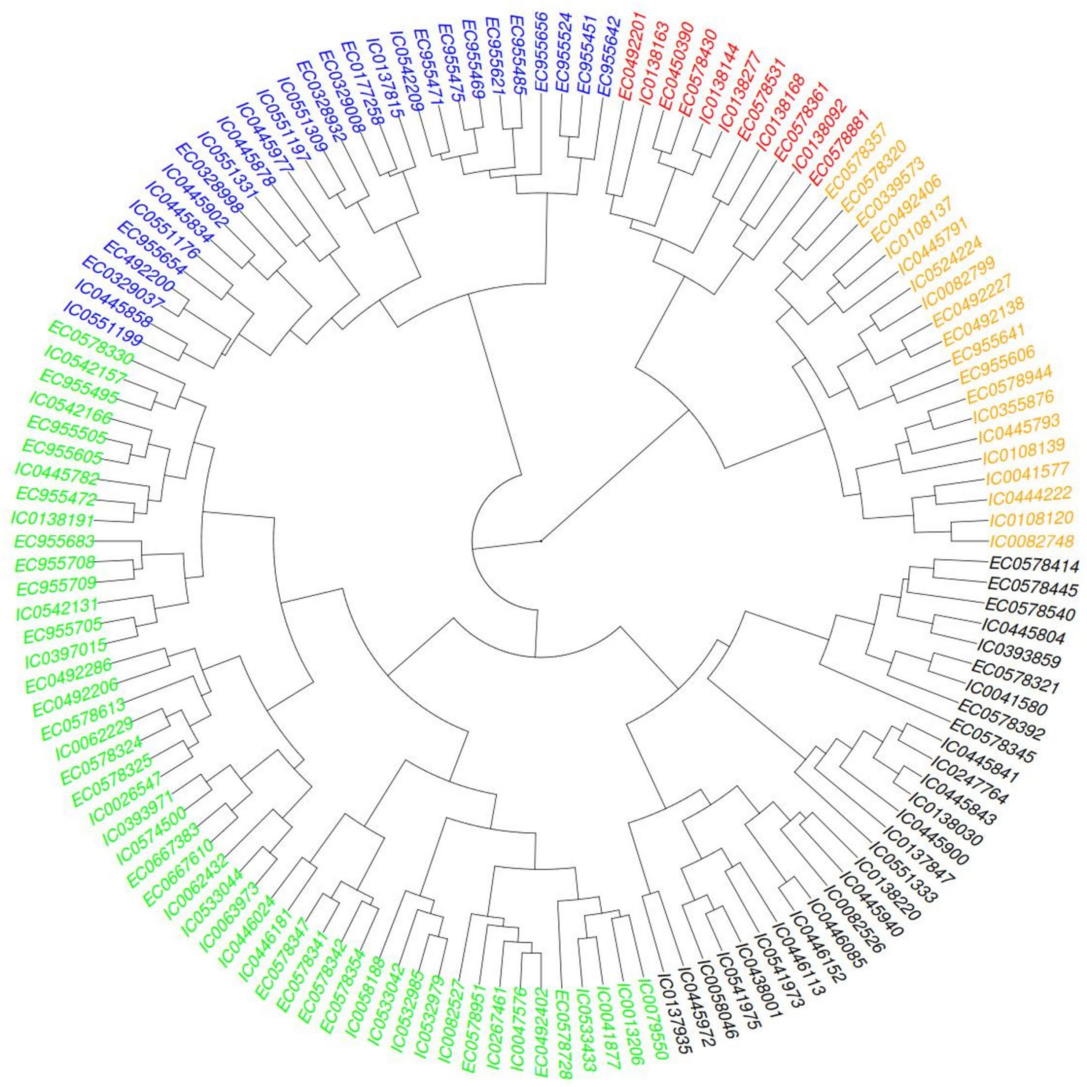
Clusters based on agro-morphological traits.

Hierarchical clustering was also performed based on agro-morphological traits a heat map provided in Figure 5 showing distribution of five major clusters so formed. Euclidean square distance of 5 was used as a distance linkage metric between groups. Figure 6 depicts the distribution of hierarchical clusters based on agro-morphological traits. Table 5 provides an insight into the biochemical traits associated with these clusters. Significant groupings were not discriminating 2-row and 6-rowed accessions.

**Table 5:** Means of biochemical traits for individual clusters (5) obtained from hierarchical cluster analysis on basis of agro-morphological traits.

Cluster	Cases per cluster	TPC	PC	B-gC	SC	AC
I	11	0.29	12.2	3.6	48.6	16.1
II	28	0.30	12.3	3.5	50.3	16.6
III	49	0.32	12.7	3.5	49.2	16.4
IV	28	0.27	12.97	3.7	49.0	16.5
V	20	0.31	11.8	3.4	51.9	17.2

The hierarchical cluster analysis of barley germplasm based on agronomic traits identifies five distinct groups with unique characteristics.

Cluster I had 11 members and was characterized with higher values for DSE (109 to 124 days) and DPM (131 to 147 days), moderate to lower values for GFT (13 to 32 days) and GY (14.47 to 131.58 g.). Other traits had assorted ranges with PH (94.4 to 137.33 cm), SL (6.33 to 11 cm), STG (21.33 to 32), GNS (18.33 to 61.33), HGW (2.09 g to 4.7 g), GWL (0.23 to 0.43).

Cluster II had 28 members characterized with moderate to lower values of GWL (0.24 to 0.38), and moderate to higher values for SL (8 to 11.3 cm), STG (23.33 to 35) and HGW (4.18 to 6.7 g). DSE (81 to 107 days), DPM (121 to 147 days), GFT (26 to 53 days), PH (87.33 to 167 cm), GNS (20.67 to 31.67) and GY (49.06 to 184.21 g) had assorted range.

Cluster III had 49 members characterized with moderate to low DSE (81 to 105 days) and DPM (120 to 143 days) and moderate to High GNS (16.67 to 71.67). Other traits exhibited assorted pattern with GFT (28 to 56 days), PH (91.83 to 132.93 cm), SL (5.17 to 10.97 cm), STG (15.33 to 30.33), HGW (2.41 to 5.58 g), GY (32.86 to 160 g) and GWL (0.25 to 0.4).

Cluster IV having 28 members was found to be characterized with moderate to high PH (100.47 to 150.67 cm), GNS (19 to 70).DSE being in the range of 74 to 111 days, DPM ranging between 126 to 143 days, GFT ranging between 30 to 67 days, PH ranging between 100.47 to 150.67 cm, SL ranging between 6.33 to 11.17, STG ranging between 15.33 to 35, HGW ranging between 3.02 g to 5.39 g, GY ranging be 46.05 g to 236.32 g, GWL ranging between 0.25 to 0.47.

Cluster V has 20 members characterized with moderate to late DSE, moderate to low GFT and low GY. DSE ranging between 90 to 107, GFT ranging between 21 to 38 days, GY ranging between 13.71 to 172.86 g. DPM ranging between 119 to 140 days, PH ranging between 82.67 to 129.67 cm, SL ranging between 5.5 to 8.73, STG ranging between 13.33 to 27.33, GNS ranging between 15 to 61, HGW ranging between 2.43 to 4.49 g, GWL ranging between 0.28 to 0.38.

The biochemical trait among these clusters had an interesting distribution. The average of the biochemical traits of these clusters as provided in Table 5 were inconclusive. However, a few within cluster associations showed emerging patterns which can be exploited for further studies.

Cluster I, a small cluster comprising of accessions with late DSE and late DPM and low GFT were also found to have low values for PC. Later maturity for northern plains means higher temperatures during maturation phase which are linked to high protein and reduced malt extract (62). These attributes are indicative of winter barley, which thrives in extended growing seasons and hill cultivation due to its late maturity and stress tolerance. Low protein is desirable trait for malting type barley.

Cluster II accessions with characterized with high STG, SL and HGW with low GWL make it important for agronomic assessments. The accessions of this group may be exploited for improving agronomic performance during breeding cycles. The members also exhibited average to low PC (around 8 to 10%) which makes it ideal for brewing related utilization as upto11% is preferred (63).

Cluster III, characterized by early DSE combined with moderate GFT, supports early maturity, prolonged grain development, and moderate to high GNS, making it well-suited for high-yield, early-harvest environments. The extended grain development coupled with early maturity likely contributes to the moderate to high SC and AC observed in this group. This is because the granule-bound starch synthase (GBSS1) enzyme is sensitive to elevated temperatures, and early maturity allows the crop to avoid high-temperature stress during the grain-filling stage (64).

Cluster IV accessions prominently showed high PH with GNS which indicate toward lodging proneness as reported in (65). Lodging is a major problem needs to be eliminated through barley breeding programs. High B-gC and high protein make this cluster suitable for human food consumption.

The cluster V comprising of accessions with late (DSE), early DPM and low GFT, with low GY and HGW suggest a balance between early and late maturity and suitability for regions needing intermediate growing seasons and quality grains. Moderate to late GFT combining with moderate to low B-gC and moderate to high AC as suggesting toward different segregants selection for these traits during breeding cycles as suggested in (17).

These insights from HCA facilitate breeding strategies tailored to specific agricultural and environmental requirements.

NIRS has been applied for screening germplasm for multiple nutrients in several crops, including sugarcane (66), cowpea (67), potato (68), Brassica (69), pearl millet (70), and maize (71). This study provided promising indications toward application of NIRS based approach for a large collection of barley germplasm and its effectiveness in obtaining a diverse set for biochemical and agro morphological traits.

The diverse panel is a valuable resource for Genome-Wide Association Studies (GWAS). GWAS in barley has been previously reported for traits such as disease resistance (72), drought tolerance (73) and elemental composition (74). The broad variation observed in agro-morphological and biochemical traits within this panel highlights its potential as an ideal candidate for GWAS analysis. Furthermore, this study can be extended to facilitate the identification of quantitative trait loci (QTL), contributing to a deeper understanding of trait inheritance and genetic architecture in barley.

While significant correlations between agro-morphological and biochemical traits were not detected, Principal Component Analysis (PCA) provided valuable insights by identifying specific accessions that showed alignment with particular traits. These accessions represent promising candidates for targeted breeding programs. By exploiting their unique trait combinations, they can serve as potential parental lines in the development of base materials for genetic improvement. This approach could facilitate the selection of desirable traits for specific breeding objectives, ultimately contributing to the enhancement of crop performance and adaptability.

## 4 Conclusion

Our results showed promising results with respect to exploitation of NIRS based approaches in research programs for mining diverse set or trait specific accessions. This approach facilitated the convergence of biochemical diversity within a large germplasm collection into a smaller subset of samples exhibiting significant biochemical variation. Besides providing a good diversty range for biochemical traits, the diversity range obtained for agro-morphological traits was also comparable to reported ranges in the barley core collection. Previous reports indicate that conventional techniques used to assess agro-morphological diversity may inadequately capture the extent of biochemical trait diversity in crops. Thus, NIRS based approaches may be integrated with the conventional techniques for developing composite core representing agro-morphological and biochemical diversity. This study also suggests GWL, i.e., grain width to length ratio as a better descriptor for plumpness especially in hulled barley, the only phenotypic trait showing relationship with grain composition. The identified trait specific accessions can be utilized for future trait-based crop improvement programs, developing mapping population and related QTL mining.

## Data Availability

The raw data supporting the conclusions of this article will be made available by the authors, without undue reservation.
